# Exploring the landscape of developmental and epileptic encephalopathies and gene research global dynamics via bibliometric study 2001–2025

**DOI:** 10.3389/fneur.2025.1733441

**Published:** 2025-12-16

**Authors:** Wen-hui Liu, Si-qi Zhang, Yan Ding, Ying Zhao, Heng Meng

**Affiliations:** Department of Neurology, The First Affiliated Hospital of Jinan University, Guangzhou, China

**Keywords:** developmental and epileptic encephalopathies, gene, bibliometric analysis, Web of Science, VOSviewer, CiteSpace

## Abstract

**Objective (background):**

The aim of this study is to analyze the current research status and future prospects of the field of developmental and epileptic encephalopathy (DEE) and gene through bibliometric methods. It aims to explore the trends and potential developments in this field.

**Methods:**

A systematic search of the DEE and gene literature from 2001 to 2025.2 was conducted using the Web of Science core collection database. Supplementary PubMed searches for this field’s clinical research trends ensured verified data comprehensiveness and methodological rigor. Quantitative analysis of co-authorship networks was performed using VOSviewer and CiteSpace tools.

**Results:**

A total of 1,022 articles related to the field of DEE and gene were included in this study, authored by 8,355 researchers affiliated with 7,238 institutions across 315 countries. United States emerged as the leading research countries in this field, with the National Institute of Health and Medical Research. Professor Ingrid Scheffer had the highest number of publications in this field, and the journal *Epilepsy* had the highest citation count. The research hotspots in this field revolved around epilepsy, mutations, epileptic encephalopathy, *de novo* mutations, and seizure.

**Conclusion:**

The research on DEE and gene is currently experiencing rapid growth. The field is expanding, and the research is becoming more in-depth.

## 1 Introduction

Developmental and epileptic encephalopathies (DEEs) are a group of heterogeneous epilepsy syndromes defined by developmental impairment, which is closely linked to both the underlying pathological etiology and the persistent epileptic process itself. Clinically, they impose a heavy burden on pediatric populations: 1 in 340 children under 16 have concurrent epilepsy and developmental impairment, while the prevalence of DEEs specifically reaches 1 in 590 children (1). This high incidence highlights the urgency of exploring DEE etiologies and advancing research to improve clinical management—particularly given that genetic mutations account for a large proportion of cases, with 112 DEE-associated genes identified to date (2), including *GABRA1* (3), *TANC2* (4), *CSMD1* (5), and *GABRB3* (6), alongside other origins like structural brain abnormalities and metabolic disorders (7). The International League Against Epilepsy (ILAE) has guided the standardized evolution of the DEE concept. In 2010, it proposed “epileptic encephalopathy” and defined “developmental encephalopathy” as age-worsening developmental delay/disability caused by underlying factors (8). A key finding—that cognitive impairment and epilepsy from the same gene are independent (some patients show pre-seizure delay or ongoing decline even with controlled seizures)—led to the formal introduction of “DEEs” in 2017: diseases characterized by drug-resistant epilepsy, abnormal EEG, and impaired brain development, encompassing both etiology-driven developmental encephalopathy and epileptic activity-related neurocognitive issues (9). In 2022, the ILAE merged DEE-related syndromes and classified them by onset age, further standardizing research and clinical practice (10).

While DEEs and gene research have grown substantially, these knowledge structure, research trends, and collaborative networks remain unexamined via systematic bibliometrics. This study addresses this gap by analyzing DEE and gene-related literature from 2001 to February 2025 using VOSviewer and CiteSpace. By mapping collaborations, identifying hotspots, and tracing evolutionary trajectories, we aim to clarify future research directions for the field.

## 2 Research methods and data sources

### 2.1 Data sources

**Figure 1:**
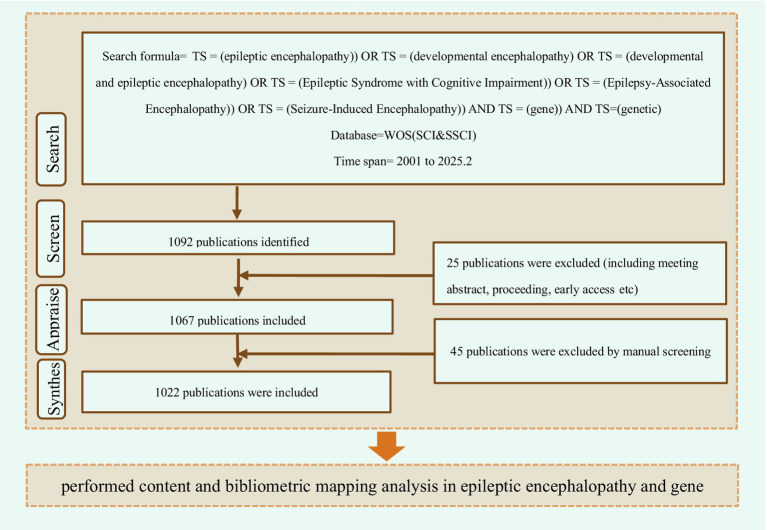
Inclusion and analysis flow chart.

The relevant articles on gene and DEEs were retrieved from the Web of Science database. Additionally, a literature search was performed through PubMed to supplement the trends of clinical research in this field, so as to ensure data comprehensiveness and methodological rigor. The search formula is “(TS = (epileptic encephalopathy)) OR TS = (developmental encephalopathy) OR TS = (developmental and epileptic encephalopathy) OR TS = (Epileptic Syndrome with Cognitive Impairment)) OR TS = (Epilepsy-Associated Encephalopathy)) OR TS = (Seizure-Induced Encephalopathy)) AND TS = (gene)) AND TS = (genetic).” This study covers all types of articles from 2001 to Feb 2025. After screening and removing duplicates, a total of 1,022 publications in the field were obtained (Figure 1). These records were written by 8,355 authors from 7,238 institutions in 315 countries and cited 25,625 references from 261 different journals.

### 2.2 Research methods

VOSviewer 1.6.19 and CiteSpace 6.3.10 were utilized to analyze the relevant literature from the Web of Science database in this study. First, a thesaurus was constructed by VOSviewer, and synonyms were merged in the literature (11). Subsequently, the data downloaded from the Web of Science was processed using CiteSpace and VOSviewer to create a new project to visualize analysis (12, 13). The parameter setting of CiteSpace was Method (LLR), Time Splicing (2001–2025), Text Processing (all term source), and Node types (choose the node we need). The parameter setting of the VOSviewer was chosen type of data, data source, and analysis and counting method. Finally, bibliometric maps were constructed by the CiteSpace and VOSviewer software, including scientific productions, authors, countries, institutions, co-cited journals, co-cited references, and keywords.

## 3 Results

### 3.1 Temporal distribution map of publications

**Figure 2:**
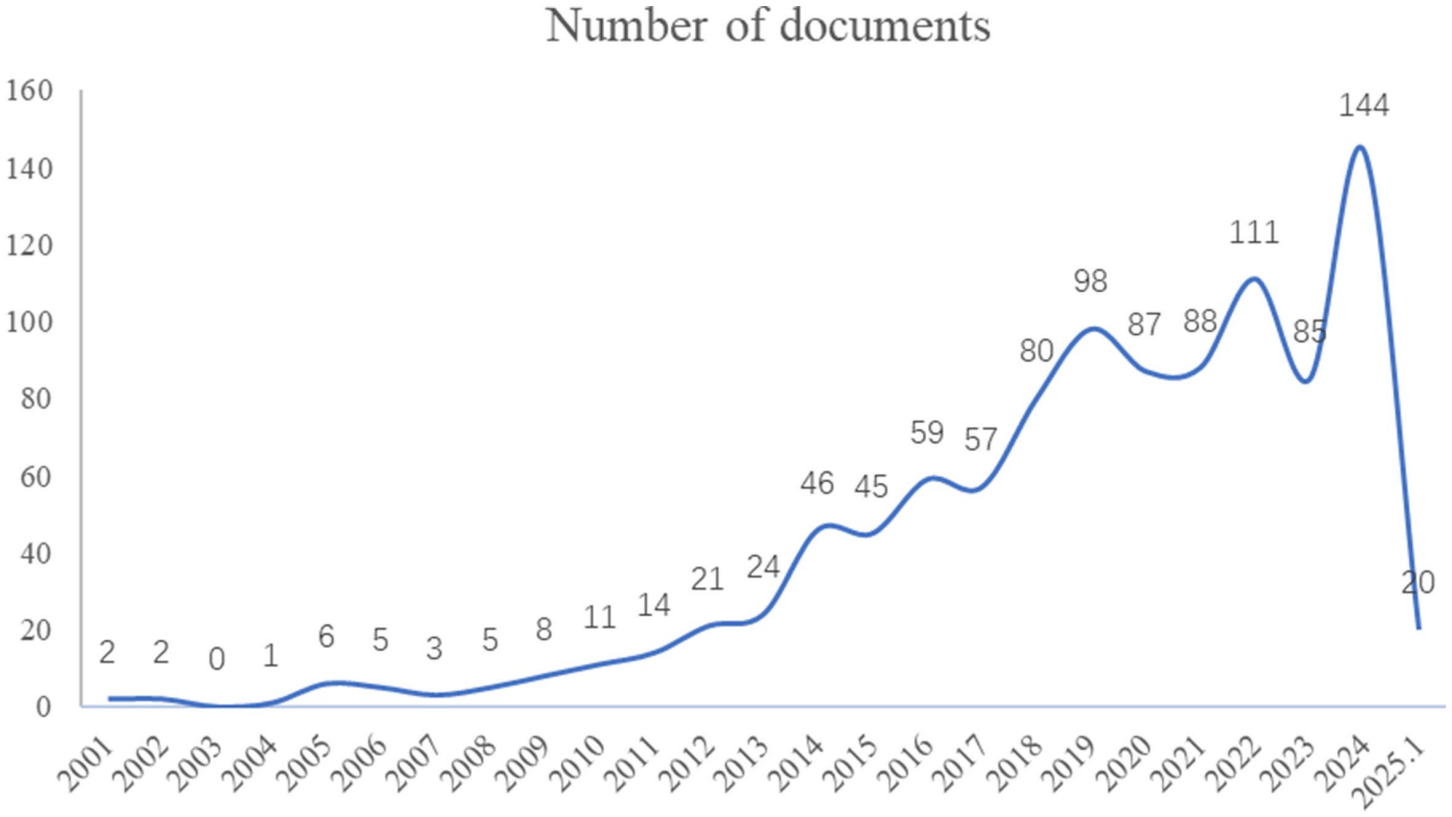
The annual analysis of research publications related to epileptic encephalopathy and gene.

The overall trend of research publications on gene and DEEs from 2001 to the present is shown in Figure 2. As the figure shows, in 2010, the International League Against Epilepsy (ILAE) first proposed the concept of epileptic encephalopathy, and the literature publication has established an annually rapidly increasing trend, reaching a peak in 2024. This indicates that the field of epileptic encephalopathy and gene received increasing attention from scholars and became a prominent research hotspot in recent years.

### 3.2 Analysis of authors

**Figure 3:**
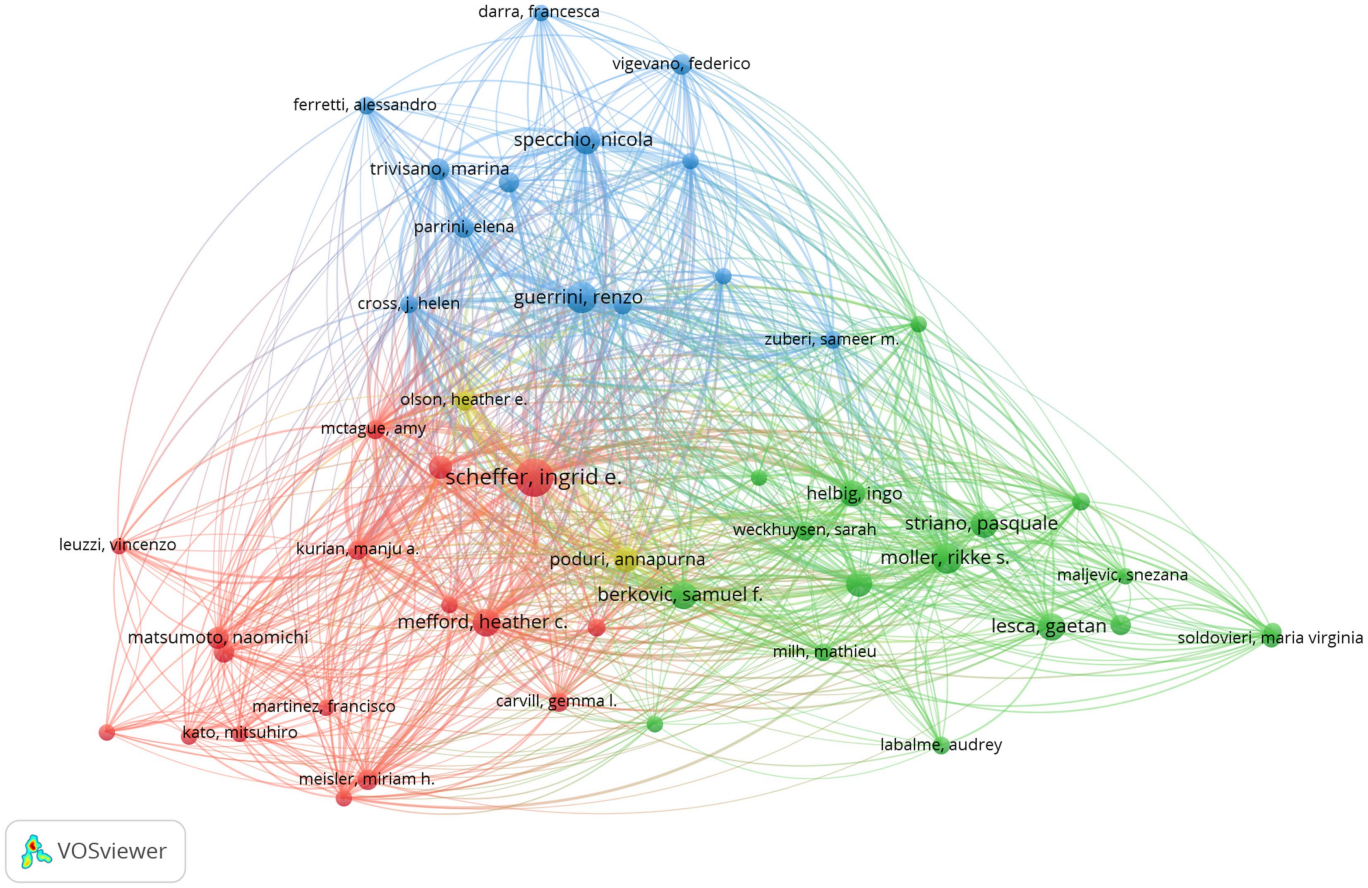
Author analysis of research on epileptic encephalopathy and gene, where circles represent authors, and lines denote their collaborations.

Bibliometric analysis of the authors can help us identify the representative scholars in the field (13). The publications were authored by 8,355 researchers in the analysis. The top five authors in terms of publications are presented in Table 1. Professor Ingrid Scheffer, from The University of Melbourne, Australia, was the highest productive author in the field. It can be seen from Figure 3 that Professor Ingrid Scheffer has close connections with each author. The result indicates that these authors have a significant influence in the field of epileptic encephalopathy and gene.

**Table 1:** Top five authors in the field of epileptic encephalopathy and gene based on publication frequency.

Author	Number of publications	Citation count	TLS
Ingrid Scheffer	34	2,490	585
Guerrini Renzo	23	1,060	310
Rikke Steensbjerre Møller	22	615	298
Nicola Specchio	18	1,058	267
Samuel F. Berkovic	18	719	267

### 3.3 Analysis of countries

**Figure 4:**
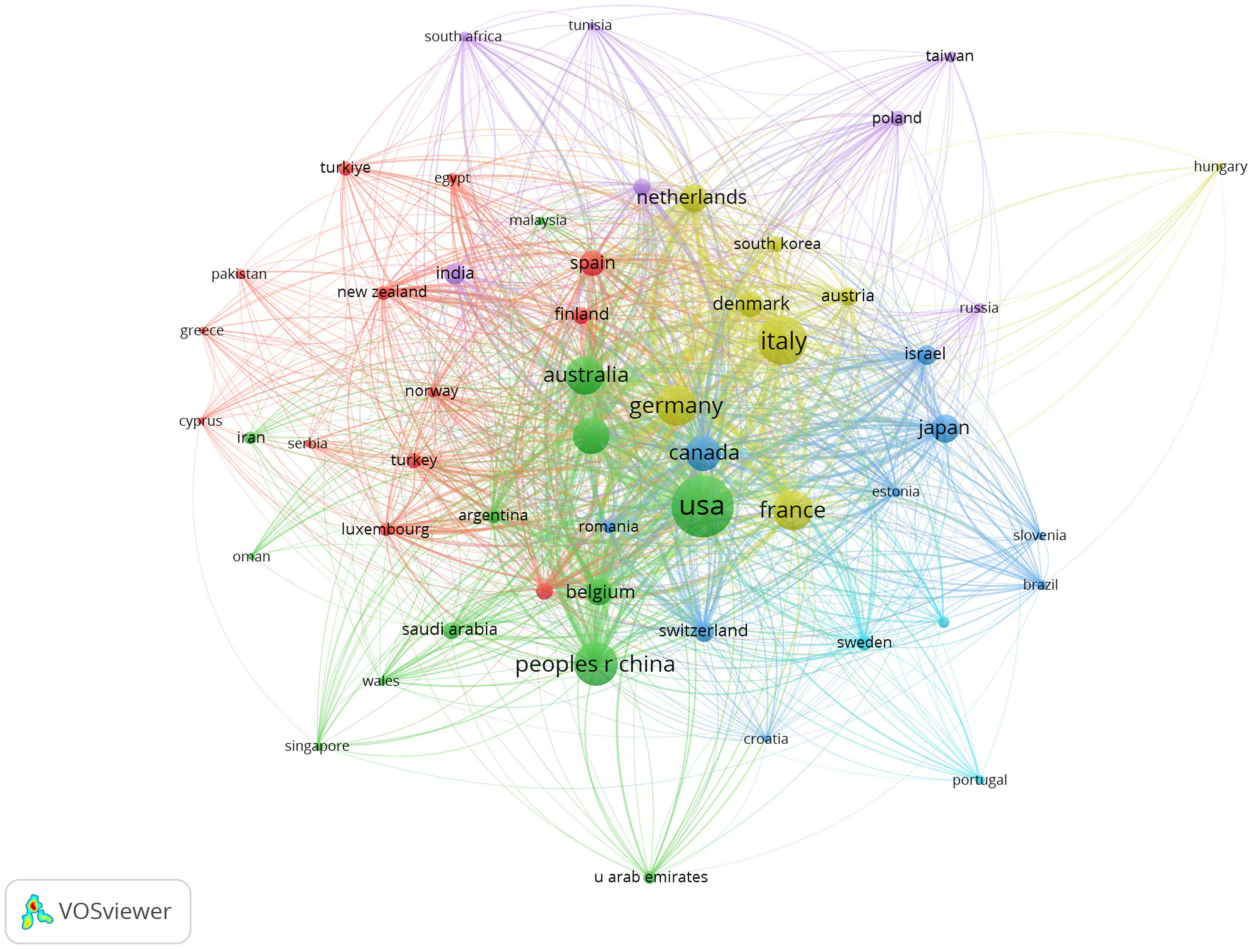
Network visualization of national collaborations in developmental and epileptic encephalopathies and gene research, where circles represent countries, and lines denote their collaborations.

In this study, the contributions of 315 countries in the field of epileptic encephalopathy and gene were analyzed. The top five countries published 901 articles, accounting for 88.16% of the total literature in this field. To gain a clearer understanding of each country’s contributions, we used VOS viewer to filter countries with five or more publications to conduct visualized analysis (Figure 4). At the same time, the top ten countries in terms of publications are listed in Table 2. The publications among countries were imbalanced. The United States (306, Centrality = 0.02), Italy (192, Centrality = 0.01), and China (153, Centrality = 0.00) had the highest number of publications, surpassing other countries by a considerable margin. This indicates that a majority of the research articles in this field originate from a few countries.

**Table 2:** Top 10 countries and institutions in developmental and epileptic encephalopathies and gene relationship research publications.

Rank	Country	Publication count	Centrality	Rank	Institution	Publication count
1	United States	306	0.02	1	National Institute of Health and Medical Research	88
2	Italy	192	0.01	2	The University of Melbourne	83
3	China	153	0.00	3	Florey Institute of Neuroscience and Mental Health	71
4	Germany	138	0.06	4	The University of London	70
5	France	122	0.04	5	Harvard University	65
6	Australia	115	0.06	6	The University College London	64
7	England	108	0.03	7	Assistance Publique – Hopitaux de Paris	60
8	Canada	98	0.09	8	Harvard Medical School	57
9	Netherlands	68	0.02	9	Royal Children’s Hospital of Melbourne	56
10	Japan	63	0.05	10	Centre national de la recherche scientifique	55

### 3.4 Analysis of institutions

**Figure 5:**
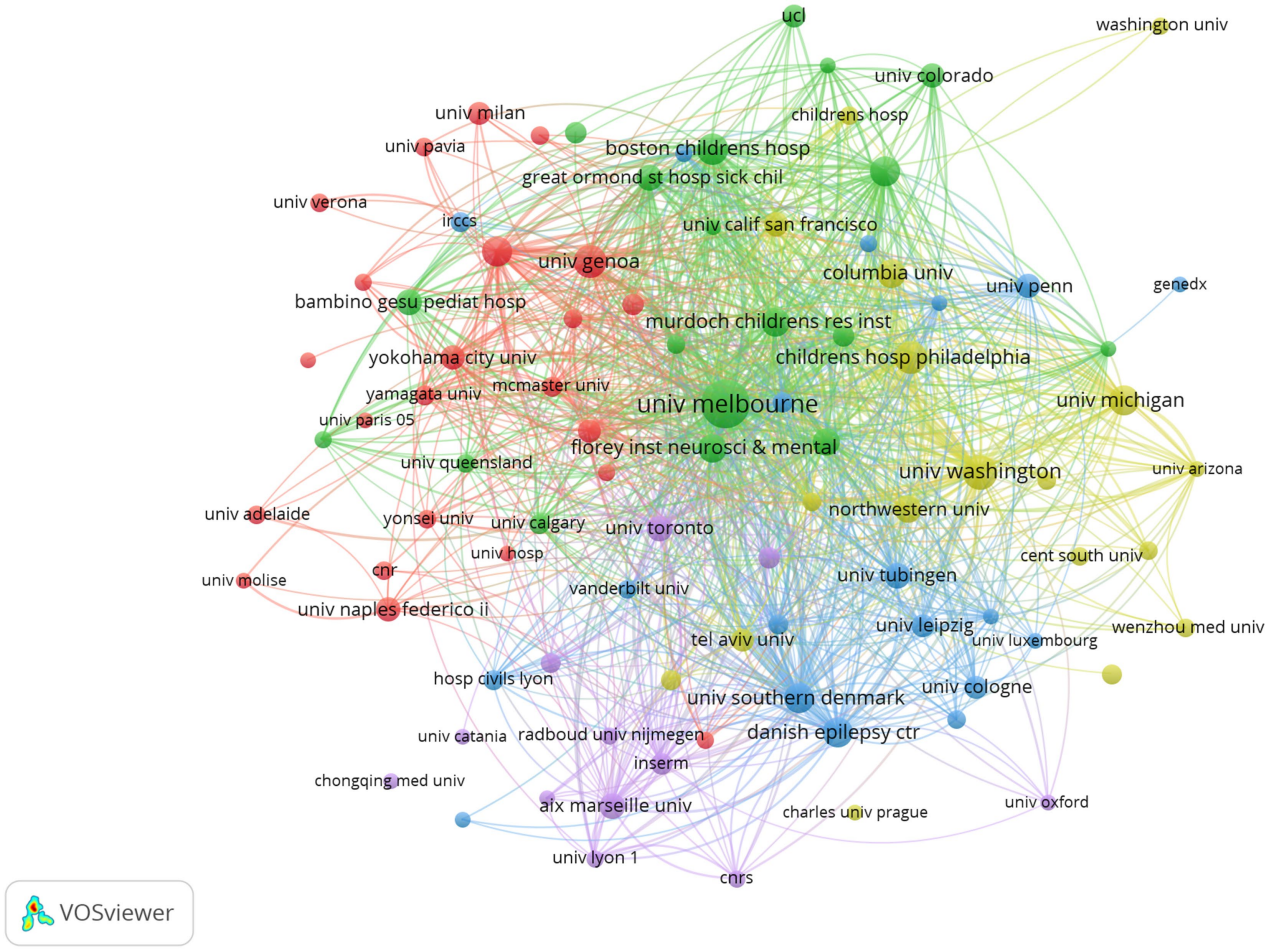
Collaboration network of institutions in developmental and epileptic encephalopathies and gene research, where circles represent institutions, and lines denote their collaborations.

Since 2001, 7,238 institutions have published literature in the field. Table 2 presents the top ten most productive institutions. The institutions with the most published were the National Institute of Health and Medical Research (*n* = 88), followed by The University of Melbourne (*n* = 83), and Florey Institute of Neuroscience and Mental Health (*n* = 71). As can be seen in Table 2 and Figure 5, the number of papers of the top institutions accounts for a relatively small proportion of total publications, which indicates that the formation of the preferential attachment effect has not yet emerged.

### 3.5 Analysis of co-citation journals

**Figure 6:**
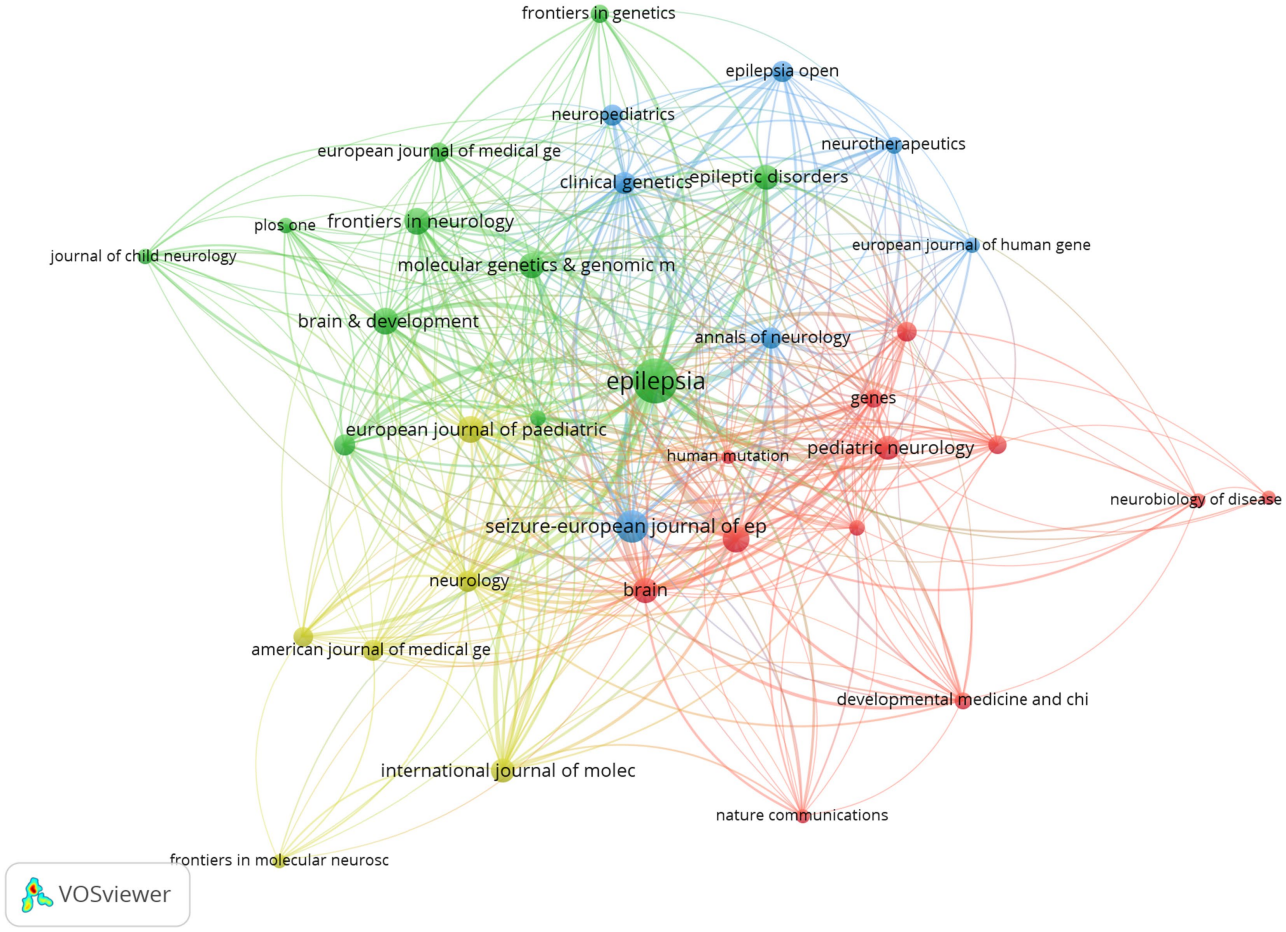
The co-citation network of journals in epileptic encephalopathy and gene research, where circles represent journals, and lines denote their collaborations.

Co-citation is the frequency with that two articles are cited together. The top 10 co-citation journals are shown in Table 3, with half of them belonging to Q1. The journal with the highest co-citation is *Epilepsy* (70, Q2, IF = 2.30), followed by *Seizure-european journal of epilepsy* (36, Q3, IF = 2.70) and *The American Journal of Human Genetics* (26, Q1, IF = 8.18). These findings show that the co-citation journals in the field of epileptic encephalopathy and gene have high quality and impact. The top 36 journals, being co-cited by at least 7 times, were used for the analysis through the VOSviewer tool. (Figure 6).

**Table 3:** Top 10 co-citation journals in developmental and epileptic encephalopathies and gene research.

Rank	Cited journals	Co-citation frequency	JCR	IF
1	*Epilepsy*	70	Q2	2.30
2	*Seizure-european journal of epilepsy*	36	Q3	2.70
3	*The American Journal of Human Genetics*	26	Q1	8.18
4	*Frontiers in Neurology*	26	Q3	2.7
5	*European journal of paediatric neurology*	25	Q1	4.8
6	*Brain & development*	25	Q1	3.8
7	*Brain*	24	Q1	10.78
8	*Epileptic disorders*	23	Q3	1.9
9	*Molecular Genetics & Genomic Medicine*	22	Q4	1.5
10	*International journal of molecular sciences*	21	Q1	4.9

### 3.6 Analysis of co-citation references

**Figure 7:**
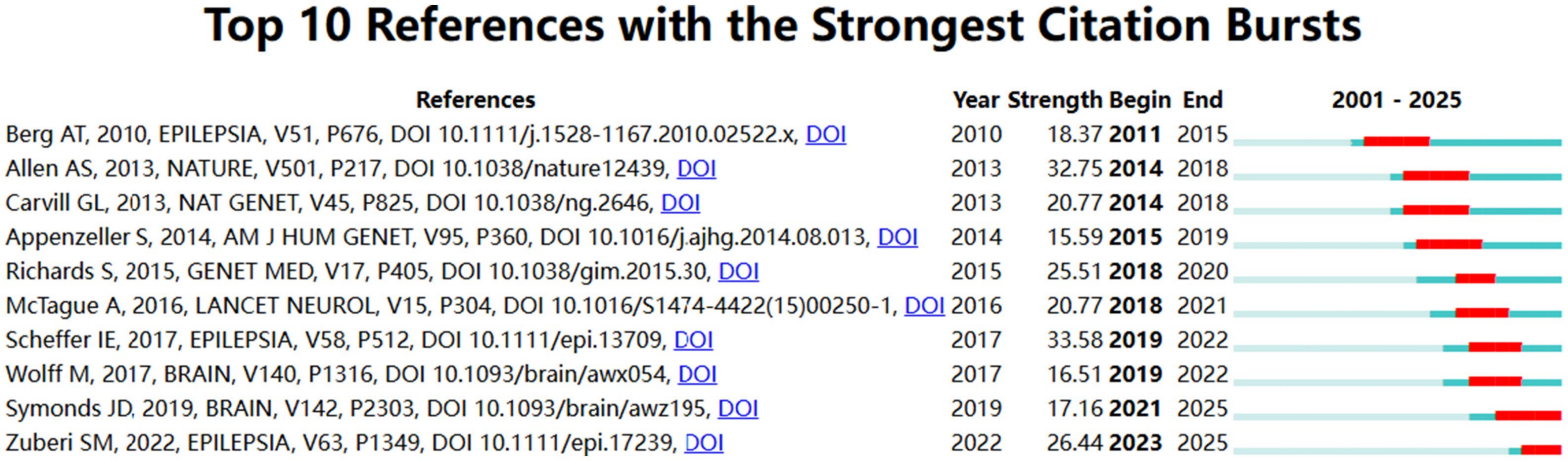
Visual analysis of literature emergence in developmental and epileptic encephalopathies and gene research. Burst strength and time duration of the top 10 references with the strongest citation bursts.

Since 2001, there are a total of 25,625 co-citation references taken into analysis in the field. The top five co-citation references were listed in the Table 4. In this article, *“De novo mutations in epileptic encephalopathies”* was written by Andrew S. Allen, and had the highest number of citations (*n* = 111). He highlighted the strong correlations between *de novo* mutations in the genes *GABRB3* and *ALG13* and epileptic encephalopathies, such as infantile spasms and Lennox–Gastaut syndrome, and demonstrated an enrichment of these mutations within particular gene sets, notably those regulated by the fragile X protein. And the top 10 co-citation references with the strongest burst strength were displayed in Figure 7, and the burst strength values ranged from 15.59 to 33.58. Among them, the reference with the highest burst strength (33.58) came from *“ILAE classification of the epilepsies: Position paper of the ILAE Commission for Classification and Terminology.”*

**Table 4:** Co-citation references.

Reference	Co-citation count	Authors
*De novo* mutations in epileptic encephalopathies	111	Andrew S Allen
Targeted resequencing in epileptic encephalopathies identifies *de novo* mutations in *CHD2* and *SYNGAP1*	111	Gemma L Carvill
*KCNQ2* encephalopathy: emerging phenotype of a neonatal epileptic encephalopathy	77	Sarah Weckhuysen
*De novo* mutations in the gene encoding *STXBP1* (MUNC18-1) cause early infantile epileptic encephalopathy	72	Hirotomo Saitsu
Revised terminology and concepts for organization of seizures and epilepsies: report of the ILAE Commission on Classification and Terminology, 2005–2009	71	Anne T Berg

### 3.7 Analysis of keyword

**Figure 8:**
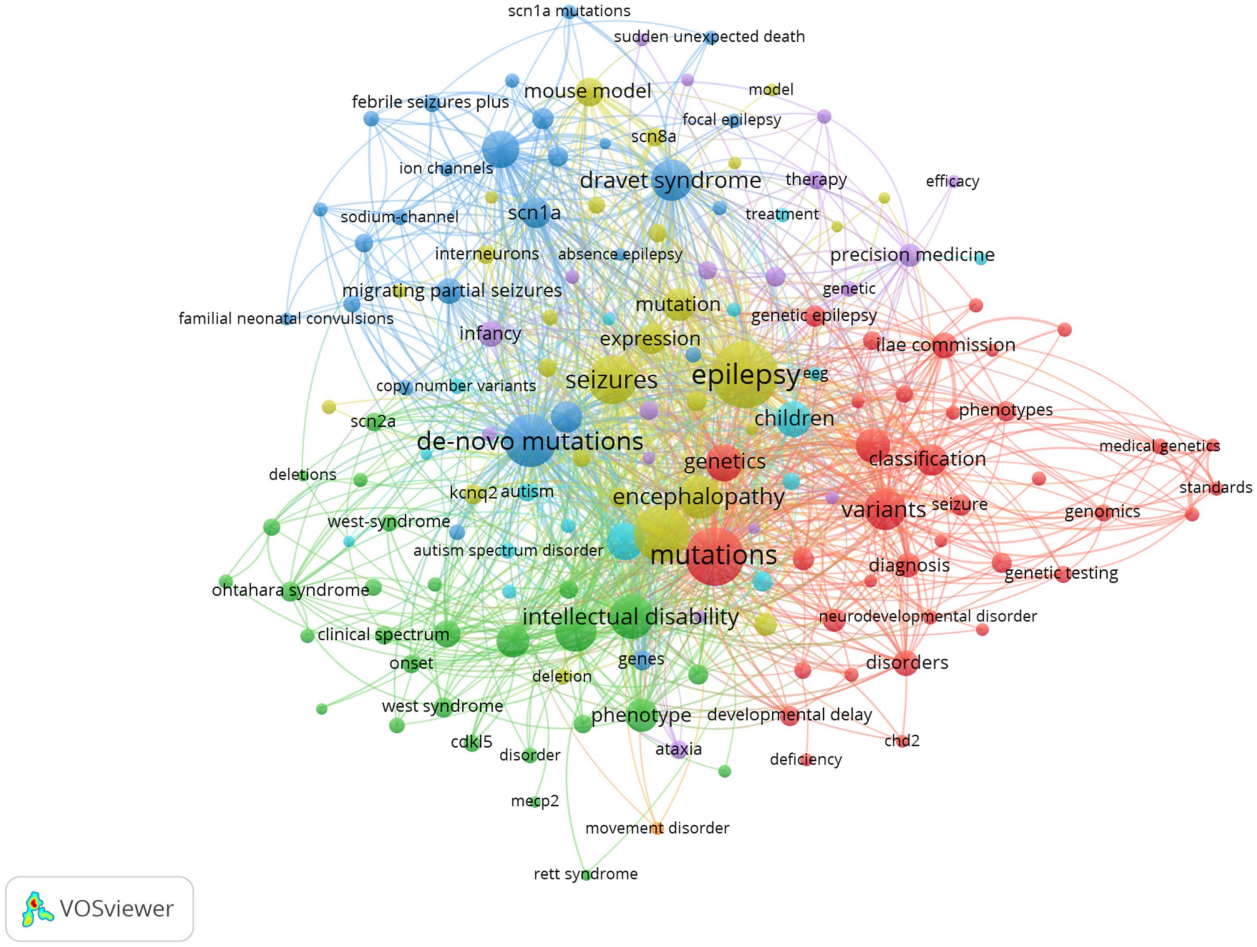
Keyword visualization map of developmental and epileptic encephalopathies and gene, where circles represent keywords, and lines denote their collaborations.

High-frequency keywords in articles reflect the hot spots of a research field (14). Table 5 lists the top 10 most frequently occurring keywords in the field. It can be seen that high-frequency keywords such as epilepsy, mutations, epileptic encephalopathy, *de novo* mutations, and seizure constitute the representative academic terminology in the field, which show the hotspots in the field. Furthermore, we selected 154 keywords that appeared with a frequency greater than or equal to 10 for visual analysis (Figure 8).

**Table 5:** Top 10 most frequently occurring high-frequency keywords in developmental and epileptic encephalopathies and gene research.

Keywords	Frequency	TLS	Keywords	Frequency	TLS
epilepsy	294	1,529	Intellectual disability	131	695
mutations	224	1,025	encephalopathy	121	627
epileptic encephalopathy	206	962	gene	115	480
*de novo* mutations	184	1,084	variants	114	676
seizure	152	884	Dravet syndrome	112	729

## 4 Discussion

As the research on the developmental and epileptic encephalopathies has deepened, scholars have increasingly focused on the relationship between developmental and epileptic encephalopathies and gene. In recent years, the number of studies in this field has grown significantly. This study quantitatively analyzed scientific outputs, countries, institutions, journals, authors, references, and keywords. Such an analysis is likely to assist in discerning the latest advancements, evolutionary trajectories, cutting - edge research hotspots, and future research trends in this area.

The temporal distribution of publications reveals a clear upward trajectory, with a marked acceleration after 2010 and a peak in 2024. This trend is closely aligned with the conceptual evolution of DEEs led by the International League Against Epilepsy (ILAE): the 2010 proposal of “epileptic encephalopathy” and “developmental encephalopathy” clarified the core pathological features of the disease, laying the foundation for targeted research The 2017 formal definition of “DEE” further integrated these two concepts, emphasizing the heterogeneity of drug-resistant epilepsy and EEG abnormalities, which standardized research objectives and promoted interdisciplinary collaboration. The 2022 ILAE classification of DEE-related syndromes by onset age further refined clinical and research frameworks, driving a surge in studies focusing on age-specific phenotypes and etiologies.

Analysis of countries and institutions reveals an uneven yet collaborative global research landscape in the field of DEE: the top five countries account for 88.16% of all publications, with the United States taking the lead—this dominance stems from strong funding support (e.g., from the U.S. National Institutes of Health), advanced technical platforms (such as gene editing and animal model development), and interdisciplinary teams integrating neurology, genetics, and molecular biology. Italy and Australia also stand out as key contributors, driven by robust clinical research networks focused on pediatric epileptic encephalopathies, exemplified by institutions like the University of Melbourne and the Florey Institute of Neuroscience and Mental Health. At the institutional level, the National Institute of Health and Medical Research ranks first in publication count, reflecting the role of public research institutions in coordinating large-scale genetic cohort studies. However, the “preferential attachment effect” has not yet fully materialized, as the top 10 institutions contribute only a small proportion of total publications—this creates opportunities for emerging institutions, especially those in low- and middle-income countries, to engage in global collaboration, a need made urgent given the global burden of DEEs (affecting 1 in 590 children under 16 years of age).

The identification of core authors further clarifies the intellectual backbone of the field. Professor Ingrid E. Scheffer led with 34 publications and 2,490 citations, reflecting her pivotal role in bridging clinical and genetic research—including contributions to DEE classification and the identification of *SCN1A* variants in Dravet syndrome, a prototypical DEE (15). Her close collaborations with other top authors have facilitated the validation of genotype–phenotype correlations, such as the link between *SCN1A* haploinsufficiency and GABAergic interneuron dysfunction (16, 17). Other key authors, such as Samuel F. Berkovic and Nicola Specchio, have focused on translational research—including the development of preclinical models for DEEs and the evaluation of non-pharmacological treatments (18–20). The collaborative networks among these authors highlight the importance of cross-institutional and cross-national partnerships in resolving the complexity of DEE genetics.

Genetic factors are the primary cause of DEE, mainly driven by monogenic variants (with *de novo* variants predominating) and supplemented by inherited patterns like autosomal dominant/recessive and X-linked inheritance. Pathogenic genes disrupt key neurodevelopmental processes, leading to imbalanced neuronal excitability and neurodevelopmental disorders. Major mechanisms include: (1) Ion transport abnormalities: variants in genes like *SCN1A* (LOF causes Dravet syndrome, GOF leads to EIDEE), *KCNQ2*, and *CACNA1A* impair transmembrane ion flux, triggering excessive neuronal excitability; (2) Synaptic transmission defects: variants in genes such as *SYNGAP1*, *STXBP1*, and newly identified *TANC2* disrupt neuronal signal communication (e.g., *TANC2* variants destabilize synapses and increase seizure susceptibility in Drosophila); (3) Signal transduction disorders (e.g., DEPDC5 in the mTOR pathway); and (4) Epigenetic dysregulation (e.g., *EP400* for chromatin remodeling, *MECP2* for transcriptional activation) (4, 15, 21, 22). These gene-mediated abnormalities collectively manifest as DEE phenotypes like epilepsy and intellectual disability.

### 4.1 Strengths and limitations

This study conducted a bibliometric analysis of research literature on developmental and epileptic encephalopathies and gene in the WOS database. However, the comprehensiveness and authority of the analysis results may be affected due to limitations in the database and language types. To enhance the reliability of the analysis, we plan to expand to other relevant databases and further investigate the research on developmental and epileptic encephalopathies in its interdisciplinary fields to improve our analysis results.

## 5 Conclusion

This study conducted a quantitative and visual analysis in the field of DEE and gene. With the help of CiteSpace and VOSviewer, we have a deeper understanding of the latest progress, evolution paths, frontier research hot spots, and future research trends in the field. And the field of DEE and gene research has received extensive attention from countries and institutions, especially the United States. Professor Ingrid Scheffer had the highest number of publications in this field, and the journal *Epilepsy* had the highest citation count. The research hotspots in this field revolved around epilepsy, mutations, epileptic encephalopathy, *de novo* mutations, and seizure. Overall, our results will help researchers understand the research trend, and provide clues for future research directions in the field.

## Data Availability

The original contributions presented in the study are included in the article/supplementary material, further inquiries can be directed to the corresponding authors.
