# Correction: Exploring the landscape of developmental and epileptic encephalopathies and gene research global dynamics via bibliometric study 2001–2025

**DOI:** 10.3389/fneur.2026.1781415

**Published:** 2026-01-20

**Authors:** Wen-hui Liu, Si-qi Zhang, Yan Ding, Ying Zhao, Heng Meng

**Affiliations:** Department of Neurology, The First Affiliated Hospital of Jinan University, Guangzhou, China

**Keywords:** developmental and epileptic encephalopathies, gene, bibliometric analysis, Web of Science, VOSviewer, CiteSpace

In the published article, an incorrect Funding statement was used: “The author(s) declare that financial support was received for the research and/or publication of this article. This study was supported by the Medical Science and Technology Research of Guangdong Province (No. A2023136).”

The correct funding statement appears below:

“The author(s) declared that financial support was received for this work and/or its publication. This study was supported by the Medical Science and Technology Research of Guangdong Province (No. A2023136) and the Science and Technology Projects in Guangzhou (No. 2024A03J0816).”

The original version of this article has been updated.

